# Public Attitudes towards and Management Strategies for Community Cats in Urban China

**DOI:** 10.3390/ani14162301

**Published:** 2024-08-07

**Authors:** Xuan Gu, Di Wu, Zilin Zhang, Guo Peng, Anru Ni, Bo Wang, Xiufan Xiong, Yujie Liu, Li Wang

**Affiliations:** 1 Department of Social Work, School of Philosophy and Social Development, Shandong University, Jinan 250100, China; nielsen@sdu.edu.cn (A.N.); emilyxiong1874@outlook.com (X.X.); yujie13627960491@outlook.com (Y.L.); echo19960112@gmail.com (L.W.); 2 Center for Animal Protection Studies, Shandong University, Jinan 250100, China; dihhshx@gmail.com (D.W.); zilin1126@outlook.com (Z.Z.); guopeng@sdu.edu.cn (G.P.); wangbo@sdwu.edu.cn (B.W.); 3 Department of Sociology, Hohai University, Nanjing 210024, China; 4 Department of Philosophy, School of Philosophy and Social Development, Shandong University, Jinan 250100, China; 5 School of Education, Shandong Women’s University, Jinan 250300, China

**Keywords:** animal welfare, attitudes towards animals, cat management, community cat, China, *Felis catus*, free-roaming cat, public attitudes, trap–neuter–return (TNR)

## Abstract

**Simple Summary:**

Community cats, who are unowned and free-roaming, are common in cities worldwide. The issue of managing community cats is widely debated in scientific research and public media, including in China. To understand public opinion on this matter, we surveyed 5382 city residents in China to ask about their attitudes towards these cats and different management strategies. The study found that a large portion of people (more than 60%) were willing to live alongside community cats and opposed trapping and killing them, and they agreed or strongly agreed with the use of the TNR method and its variations. The study also found that men or those with lower education or incomes were more inclined to support trap-and-kill and doing nothing as management methods for community cats. In contrast, females or those with higher incomes and education levels had more positive attitudes towards the cats and were more inclined to oppose inaction and support the TNR method for managing the cat population. Based on these findings, we discussed the implementation of TNR with adoption programs in urban Chinse communities and the need for educational campaigns to promote humane and effective cat management strategies. By understanding public attitudes, policy makers, educators, and urban residents can develop better solutions that address both community concerns and the welfare of community cats, ultimately contributing to improved urban cat management in China.

**Abstract:**

Managing community cats in urban China is a contentious and emerging issue, with debates centering on the most effective and humane approaches. This study aimed to investigate public attitudes towards community cats and various management strategies. A survey was conducted involving 5382 urban residents in China. Their attitudes towards the positive and negative roles of community cats in urban areas and their support for different management methods were examined, including trap-and-kill, taking no action, centralized management, and trap–neuter–return (TNR) and its variations. Results indicated that 63% of participants were willing to coexist with community cats, 71% opposed trap-and-kill, and 61% agreed or strongly agreed with the TNR method and its variations. Older residents or those with higher incomes were more likely to support coexistence with community cats. In contrast, younger or lower-income residents were more likely to support non-coexistence. Residents in first- or second-tier cities (e.g., Beijing, Hangzhou, and Jinan Cities in China) were more inclined to support trap-and-kill and less likely to support coexistence than their counterparts in fourth-tier cities (e.g., county-level cities in China). Moreover, those with lower education or incomes were more supportive of trap-and-kill and taking no action as the methods to manage community cats than those with relatively higher education or incomes. Those with higher incomes held more positive attitudes towards community cats and were more supportive of TNR and its variations than their counterparts with lower incomes. Males were more inclined to support trap-and-kill and taking no action and less inclined to support centralized management and TNR than females. The implications of the findings on TNR with adoption programs in urban China are discussed. These novel findings underscore the need for targeted educational campaigns to promote humane and effective management strategies, addressing public concerns and community cats’ welfare. The study’s insights are critical for informing policy and improving community cat management in urban China.

## 1. Introduction

Domestic cats (*Felis s. catus*) are the most popular companion animals in the world [1,2,3]. In addition to the population of domestic cats as companion animals, a significant number of unowned, free-roaming domestic cats frequently interact with humans [4,5,6]. A variety of terms and definitions have been proposed to describe these cats [7], such as feral cats, unowned cats, outdoor cats, or free-roaming cats. In this study, the term “community cats” is employed, which is widely used to denote unowned, free-roaming cats regardless of their sociability [6,7,8]. This term is gaining popularity in part because it implicitly recognizes cats as a commensal species that many residents value [6]. It also advocates for the community to take social responsibility for managing and caring for them [9].

The overpopulation of community cats represents a pressing global issue [10,11,12], with estimates suggesting tens of millions in China alone [13]. This overpopulation is not solely a result of natural reproduction processes but also human actions, as community cats can originate from existing populations or unneutered lost and abandoned pets [12,14]. The concerns brought about by overpopulation include noise and hygiene nuisances [4], public health threats [15], wildlife predation [16], and the threatened welfare of the community cats [17]. In the field of scientific research, the places and roles of community cats in contemporary societies and urban ecosystems are contentious issues [2,6]. For example, some researchers have criticized studies of cat’s predation for making inflated or false claims about the real impact of cats on wild animals in urban areas [18,19]. They have highlighted the roles of community cats in rodent management and noted that anthropogenic activities threaten biodiversity more than feline behaviors [20]. Meanwhile, advocates for and studies of coexistence with community cats have been accused of using biased information to cloud scientific findings [21,22]. In the context of conflicting scientific evidence and conclusions, public perception and attitudes play a pivotal role in the formulation of policy and the practical management of community cats.

Given the paucity of empirical research on public attitudes towards community cats and their roles in urban communities in China, the first objective of this study is to ascertain residents’ attitudes towards community cats, including their views on coexistence, positive interactions, and the perceived roles of community cats in urban ecology. For example, to capture concerns about the roles of community cats in rodent control, we developed three items on an Attitudes towards Community Cats Scale and asked participants whether they agreed or disagreed with the following statements: community cats control rat populations and reduce the spread of disease; utilizing community cats for ecological rodent suppression is better than dropping rodenticides; and community cats can protect old buildings and artifacts from rats.

The controversial roles of community cats in urban areas have led to significant debate and emotional issues surrounding community cat management. Approaches to management methods vary and may involve improving the cats’ welfare [23], reducing the cats’ potential wildlife predation [16,24], and testing optimal methods to control the cat population [25,26]. While there is a consensus on the need to reduce their numbers, there is often a need for more consensus on the most effective methods of achieving this goal. This lack of consensus has given rise to high-profile debates over community cat management in various locations, including countries such as Australia [27,28] and China [19,29]. Thus, the second objective of this study is to examine public attitudes in China towards different community cat management approaches. Our study can contribute to an understanding of effective and acceptable community cat management methods and foster a sense of shared understanding and agreement.

Research and public debate have increasingly focused on lethal and non-lethal methods of community cat management [8,11]. In addition to non-surgical contraception and regulating the source of cats [30], four general methods for managing community cats are typically discussed or adopted. We examined these four methods in this study. The first is a “wait and see” or “do nothing” strategy, whereby populations are allowed to remain unmanaged [24]. This method, historically employed, is still applied in some areas, although it is often considered ineffective [26,31]. Other methods have been developed to address community cat populations. The traditional strategy involves the trapping and killing of cats, either on-site or by removing them for euthanasia. Considering cost, efficiency, public support, and ethical concerns, neither eradication nor trap-and-kill effectively addresses the challenges [8]. The trap-and-kill method also hurts cat caregivers’ well-being [17].

The third method involves trapping cats and subsequently transferring them to animal shelters or bases for centralized management. In North America, hundreds of millions of cats have been impounded and euthanized in animal shelters, and billions of dollars have been invested in such programs [11]. Two types of animal shelters or bases in China serve two distinct purposes. They can function as sanctuaries that permanently house unowned dogs and cats until the animals’ natural lives come to an end. Alternatively, they can serve as temporary holding facilities for unowned animals, pending the implementation of lethal action.

The fourth method, trap–neuter–return (TNR), has been increasingly adopted by non-profit humane organizations and animal welfare advocates. TNR involves capturing, neutering/spaying, vaccinating, and returning cats to their original locations [7]. Many programs combine TNR with adopting socialized cats and kittens or ear-tipping (e.g., removing one cm of the left ear to indicate sterilization) [32,33]. There are several variations of TNR, including trap–neuter–vaccinate–return (TNVR), which emphasizes the vaccination component [34]. In order to ensure optimal welfare, it is recommended that neutered cats be returned to their original territories. If this is not feasible, an alternative environment with adequate food may be used, designated as trap-neuter-release (TNRel) [7]. The return-to-field (RTF) method is analogous to the TNR, TNVR, and TNRel methods in that it involves the neutering/spaying, vaccination, and return of cats. However, the RTF method is a shelter-based rather than a community-based approach. It is designed for cats designated as “strays” upon admission to the shelter [34]. In the current study, we designated the TNVR, TNRel, and RTF as variations of TNR. The two main aims of TNR and its variations are to reduce the number of cats who would otherwise likely be killed and to reduce community cat populations.

Currently, most TNR programs in China are carried out by volunteers or non-profit organizations. They are primarily concentrated in communities of first-tier and second-tier cities, including Beijing, Shanghai, Shenzhen, Guangzhou, and Hangzhou [35,36,37,38]. In China, an urban community has a clearly defined territory, often demarcated by fences or walls, and constitutes a social collective composed of people residing within the bounded district [39,40]. Volunteers, working individually or as a group, implement TNR programs to control community cat populations within communities, sometimes supported by grassroots government organizations like residents’ committees [35,37]. Such programs encompass neutering/spaying, vaccination, deworming treatments, and returning the community cats to their original location (i.e., the original community where the cats were captured and previously lived, rather than returning them to the streets or the wild). Some TNR programs involve adopting the cats [36,38] and responsible feeding and regular monitoring of the cats’ health after TNR [35,41].

In formulating policies and implementing management methods, public opinion is an essential component that cannot be ignored. Thus, the second objective of this study is to investigate Chinese attitudes towards different community cat management methods. We collected data on four management methods: (a) trap and kill, (b) taking no action, (c) centralized management (sending to animal shelters or bases), and (d) TNR and its variations. We expect the findings will inform legislation on cat management, promote community cat welfare, and identify best practices for managing community cats in urban China.

The data presented are part of the “Social Research Project on Community Unowned Cats in Urban China” conducted from mid-November 2021 to early February 2022. We developed a questionnaire to address two main issues: (a) the perceived health of community cats and the quality of human–cat relationships in urban communities and (b) public attitudes towards community cats and methods for their management. The results of the first issue were reported by Gu et al. [6]. This study concentrates on the second issue and mainly explores the following three questions: What are public attitudes towards community cats in urban areas, such as the potential values and problems associated with the cats? What are public attitudes towards methods of managing community cats in urban areas? How do city and community types, as well as residents’ socio-demographic variables, predict these attitudes?

## 2. Methods

### 2.1. Sampling and Participants

We used a stratified sampling method to recruit participants and defined three strata: provinces/regions, cities, and urban communities. Based on economic development and geographical representation, we selected Beijing City, Zhejiang Province, and Shandong Province in the first strata. Then, the cities within the provinces were classified into different city tiers. The city tier classification, widely applied to urban and regional studies in China [42], was used to categorize the cities into first-, second-, third-, and fourth-tier cities. These city tiers are distinguished by their GDP and population size. In the current study, Beijing was selected to represent the first-tier cities. The second-tier cities included the provincial capitals of Zhejiang and Shandong Provinces, which are Jinan City and Hangzhou City. The third-tier cities were prefecture-level cities, while the fourth-tier cities were county-level cities or counties in the two provinces.

The survey employed a combination of online and offline methods for the distribution of questionnaires. The online survey was hosted on the Wenjuanxing platform, a survey service provider in China. Accessible via QR codes were distributed in WeChat groups of the residents within a community. A distinct link was provided to each community to facilitate the monitoring of data collection, thereby ensuring that each smartphone was permitted to yield a single response. Participants were assured anonymity throughout the process, entering a unique password for survey completion without disclosing any personally identifiable information. For those less familiar with smartphones or reluctant to use online forms, such as older adults, a paper-based alternative was made available. We ensured diversity in the sample and prevented sample bias by including residents of different ages, genders, and residential areas. The participants were paid via WeChat Pay Red Packets and received a random amount with an average of CNY 10 for online completions and approximately CNY 10 in gifts for those who opted for the paper-based questionnaire.

A total of 5382 participants were included in the data analyses. The sample comprised 2848 females and 2534 males, aged between 18 and 85, with a mean age of 41. Participants resided in 26 cities and 52 urban communities. Before their participation, all participants provided informed consent. They were informed that participation was voluntary and their responses would be anonymous and confidential. Gu et al. [6] reported the detailed sampling procedure and socio-demographic information of participants.

### 2.2. Measures

We developed a Chinese questionnaire and conducted pilot tests to evaluate and improve the questionnaire and the data collection process. To avoid any potential influence of varying designation for felines on the participants, we did not use “stray cats” or “community cats”. Instead, we employed a more impartial designation, referring to them as “unowned cats in a community” in the questionnaire. The questionnaire items can be found in Appendix A.

#### 2.2.1. Socio-Demographic and Community Variables

The socio-demographic and community variables in the current study align with those outlined in the work of Gu et al. [6]. The socio-demographic variables included gender, education level, age, employment status, annual income in the prior year, and marital status. Community-level variables included distinctions concerning city and community types. The community type was defined based on the boundaries of the community and the characteristics of the different housing properties.

Nominal variables were transformed into dummy variables for regression analyses. This process entailed creating N-1 dummy variables for a variable with N categories, with the most prevalent category designated as the reference group (and assigned a value of 0).

#### 2.2.2. Willingness to Coexist with Community Cats

Participants reported their overall attitudes towards community cats by selecting one of the five options: (a) I am willing to see unowned cats regularly in the community, hoping that they will become part of the scenery and community life and be treated well and managed appropriately in accordance with their positive roles; (b) I am relatively willing to see unowned cats in the community in the hope that they can be managed scientifically; (c) I have not considered this issue; (d) I am less willing to see unowned cats in the community, but disagree with the community using extreme methods to manage unowned cats; (e) I am totally unwilling to see unowned cats in the community. The responses “a” and “b” were grouped as “willingness to coexist,” while responses “d” and “e” were grouped as “unwillingness to coexist.” The response “c” was recoded as missing data.

#### 2.2.3. Awareness of Community Cats as an Unsolved Issue and Its Urgency

Participants were asked whether they thought community cats had become an issue that needed to be solved. If they answered yes, they proceeded to respond on a 5-point Likert scale to indicate the degree of urgency of the issue, with 5 being the most urgent and 1 being not urgent at all.

#### 2.2.4. Attitudes towards Community Cats Scale (ACCS)

A 5-point Likert scale with 10 items was developed to gauge residents’ attitudes towards community cats in urban communities. The items cover community cats’ positive and negative roles from the perspective of urban ecology and public health, including rodent management, bird predation, nuisance behavior, and potential threats to public health. While these roles may be debatable in academia, the items reflect the growing scientific discourse and public concerns about community cats. The items also address the human–animal bond, particularly the mutual interactions between community cats and humans.

Participants indicated the degree to which they agreed or disagreed with each statement, from 1 = strongly agree to 5 = strongly disagree. To generate an overall score of ACCS, the items relating to positive attitudes were reverse-scored, and then the mean of the ten items was computed. A higher average score of ACCS represents a more positive attitude towards community cats. The Cronbach’s alpha coefficient of ACCS was 0.8 in the current study, indicating high internal reliability.

#### 2.2.5. Attitudes towards Management of Community Cats

Participants expressed their attitudes towards a lethal method, i.e., trapping and killing community cats, on a 3-point Likert scale by selecting one of the three options: support, oppose, or neither support nor oppose. The responses were recoded with support = 3, neutral = 2, and oppose = 1. A higher score indicated higher support for trap-and-kill.

Except for trap-and-kill, the other three strategies for managing community cats were measured on a 5-point Likert scale from 1 = strongly agree to 5 = strongly disagree. The three types of management strategy are (a) doing nothing, i.e., taking no action; (b) centralized management, in which cats are sent to shelters or stray animal bases after being captured; and (c) TNR and its variations: neutering/spaying and vaccinating the cats after capture or treating, fostering, and returning them to their original location or otherwise rehoming them, as appropriate. The scores of the three items were reversed, with a higher score indicating higher support for a specific management strategy.

### 2.3. Statistical Analyses

Multilevel regression modeling was employed because participants were nested within communities. Predictor variables at two levels were analyzed: socio-demographic characteristics of participants at Level 1 and community variables at level 2 in the regression models. Instead of adding different cities at a third level, two-level models were employed because multilevel logistic modeling requires a minimum of 50 level 1 and 40 level 2 units, which are necessary to estimate the effects accurately [43].

We fitted multilevel logistic regressions to examine the associations between community and socio-demographic variables and the binary outcome variables, including willingness to coexist with community cats (yes = 1, no = 0) and awareness of community cats as an unsolved issue (yes = 1, no = 0). Additionally, we fitted multilevel linear regressions to examine the associations between community and socio-demographic variables and the continuous outcome variables, including attitudes towards community cats as measured by ACCS and attitudes towards the management of community cats. Multilevel linear regression models are required to meet five assumptions: (1) there is a linear relationship between the predictor variables and the outcome variable, (2) level 1 residuals and predictors are independent, (3) level 2 residuals and predictors are independent, (4) residuals at different levels are unrelated, and (5) there is homoscedasticity [44].

Statistical analyses were performed using SPSS 29 (IBM Corp, Armonk, NY, USA), and significance was set at *p* < 0.05.

## 3. Results

### 3.1. Descriptive Statistics

Table 1 presents the descriptive statistics of attitudes towards coexistence with community cats, community cats as unsolved issues, and management strategies for community cats. A predominant proportion of participants, 63% (N = 3412) demonstrated a willingness to coexist with community cats, and 14% (N = 774) expressed reluctance. This trend suggests that most residents are optimistic about cohabiting with community cats. Nevertheless, 22% (N = 1195) of the sample demonstrated an unclear stance regarding coexistence with community cats.

When participants reported they often or occasionally saw community cats, they continued to answer whether they thought community cats were an unsolved issue. Therefore, the sample sizes were adjusted to N = 4621 when the awareness of community cats as an unsolved issue was analyzed. Among the participants, 57% (N = 2644) perceived community cats as an issue awaiting resolution, and 43% (N = 1977) did not. The participants (N = 2644) who perceived the community cats as an unsolved issue continued to rate the urgency on a 5-point Likert scale. The mean score of urgency was 3.4, with a standard deviation of 1.0. The mean score suggests that cat-related concerns received acknowledgment of urgency for more than half of the residents.

Regarding attitudes toward the trap-and-kill method of cat management, 71% (N = 3836) of participants expressed opposition to it. Meanwhile, 5.1% (N = 272) indicated accepting it as an approach. The rest, 24% (N = 1274) of the participants, expressed a neutral attitude towards the lethal method. The findings suggest that most participants preferred a non-lethal method, while a minority supported a lethal one.

The option of taking no action received relatively low endorsement and acceptance. In contrast, centralized management and TNR and its variations garnered broader support and acceptance, suggesting a tendency among most participants to endorse corresponding action plans. Nevertheless, the marginal differences in mean scores and percentages between these two strategies indicate that participants did not prefer one to the other.

Table 2 presents the items and descriptive statistics of ACCS. The average score for the ten items of ACCS was 3.4, with a standard deviation of 0.6. The mean score suggests that participants’ positive attitudes towards community cats were above medium level and not exceptionally high. Overall, participants affirmed the positive contributions of community cats to urban ecosystems and acknowledged their efficacy in rodent control, enriching urban ecology, and fulfilling emotional needs. However, participants raised concerns such as sleep disruption caused by community cats’ loud vocalizations in their breeding seasons, alongside worries about potential adverse effects on human health. They did not express serious concerns about threats to safety posed by community cats to humans or other species.

### 3.2. Multilevel Logistic Regression

Table 3 presents the results of logistic regression models when the outcome variables are, separately for each model, whether they were willing to coexist with community cats and whether community cats were perceived as an unsolved issue. The answers “no” to the above outcome variables were set as references. Level 1 variables (i.e., participants’ socio-demographic variables) and level 2 variables (i.e., city type and community type) served as predictor variables.

When the outcome variable was the willingness to coexist with community cats, we considered it a binary variable with the choice of “I have not considered this issue” as missing data. Therefore, the sample size was adjusted to N = 4187. Model 1, employing “willingness to coexist with community cats” as the outcome variable, was significant, *F* (24, 4162) = 4.2, *p* < 0.001. Older participants were more likely to support coexistence with community cats than younger participants. Participants with annual incomes between CNY 100,000 and CNY 200,000 or those with incomes exceeding CNY 200,000 were, respectively, 1.4 or 1.8 times more likely to support coexistence with community cats compared to those earning less than CNY 50,000. Unmarried participants were 1.9 times more likely than married participants to support coexistence. Regarding regional differences, participants from second-tier cities were 0.6 times less likely than those from fourth-tier cities to support coexistence with community cats.

Model 2, using “awareness of community cats as an unsolved issue” as the outcome variable, was significant, *F* (24, 4596) = 3.6, *p* < 0.001. Older participants were more likely than younger participants to perceive community cats as an issue requiring resolution. Relative to married participants, unmarried participants were 0.7 times less likely to perceive community cats as an issue. Residents in first-tier cities were 0.6 times less likely to perceive community cats as an issue than those in fourth-tier cities.

In summary, the findings of multilevel logistic regressions indicate that older residents were more likely to perceive community cats as an unsolved issue and were more willing to coexist with them than younger residents. High-income residents were more likely to coexist with community cats than low-income residents. Compared to residents in four-tier cities, those in first-tier cities were less likely to perceive community cats as unsolved issues, and those in second-tier cities were less likely to be willing to coexist with community cats.

### 3.3. Multilevel Linear Regression

Table 4 presents the results of multilevel linear regression models with attitudes towards community cats and each management strategy of community cats as separate outcome variables. As in the multilevel logistic models, the predictor variables in multilevel linear models were categorized into levels 1 and 2. The assumptions of multilevel linear regression models were met.

The scores of the ten items of ACCS were averaged, and higher average scores indicated more positive attitudes towards community cats. With “attitudes towards community cats” as the outcome variable, model 3 showed significance, *F* (24, 5357) = 2.9, *p* < 0.001. Compared to participants with an annual income below CNY 50,000, those earning between CNY 100,000 and CNY 200,000 or over CNY 200,000 held more positive attitudes towards community cats. Unmarried participants exhibited more positive attitudes towards community cats compared to married participants.

Using the “trap and kill” strategy of cat management as the outcome variable, model 4 was significant *F* (24, 5357) = 6.0, *p* < 0.001. Males showed greater support for trap-and-kill than females. Participants with lower levels of education (e.g., elementary school, junior high school, or high school) more strongly agreed with trap-and-kill than participants with university or college education. Those with annual incomes between CNY 100,000 and CNY 200,000 or above CNY 200,000 more strongly disagreed with the lethal method than those earning less than CNY 50,000. Participants in first- or second-tier cities demonstrated higher support for the lethal method than their counterparts in fourth-tier cities.

Using the “take no action” strategy of cat management as the outcome variable, model 5 was significant, *F* (24, 5357) = 3.2, *p* < 0.001. Compared to females, males exhibited higher support for doing nothing. Participants with lower education exhibited higher support for taking no action compared to those with college or university education. Participants engaged in household duties or retired were more opposed to doing nothing than those fully employed. Participants with an annual income between CNY 50,000 and CNY 100,000 were more opposed to taking no action than those earning less than CNY 50,000. Unmarried participants were more opposed to doing nothing than married participants.

Using the “centralized management” management strategy as the outcome variable, model 6 was significant, *F* (24, 5357) = 4.1, *p* < 0.001. Males more strongly opposed centralized management than females. Participants with a primary school education or below supported this method more than those with a college or university education. Regarding regional differences, participants in first-tier cities strongly opposed centralized management more than those in fourth-tier cities. Participants residing in campus communities strongly supported centralized management more than those in ordinary commercial housing communities.

Using the “TNR and its variations” management strategy as the outcome variable, model 7 was significant, *F* (24, 5357) = 3.1, *p* < 0.001. Males more strongly disagreed with this method than females. Participants with an annual income between CNY 100,000 and CNY 200,000 more strongly agreed with the TNR method than those earning less than CNY 50,000.

In summary, the findings of multilevel linear regressions demonstrated that males more strongly agreed with trap-and-kill and taking no action, while they more strongly disagreed with TNR and centralized management methods than females. Those with lower levels of education were more inclined to agree with the option of trap-and-kill, taking no action, or centralized management than those with a university or college education. Those with relatively higher incomes exhibited more positive attitudes towards community cats and higher agreements with the TNR management method, and they disagreed more strongly with trap-and-kill and taking no action than residents with relatively lower incomes. In comparison to married residents, unmarried residents exhibited more favorable attitudes towards community cats and more strongly disagreed with taking no action.

## 4. Discussion

This study aimed to explore urban Chinese residents’ attitudes towards community cats and different management strategies. By surveying 5382 individuals, our findings highlight significant trends and socio-demographic influences on these attitudes. The novel findings are crucial for informing policy decisions and enhancing community cat management practices in urban China. Furthermore, the Attitudes towards Community Cats Scale (ACCS), which was developed in this study, provides a valuable foundation for further validation of its reliability and for examining the public attitudes and perceptions regarding the roles of community cats in urban areas.

### 4.1. TNR Combined with Adoption: A Suggested Method of Managing Community Cats in Chinese Urban Communities

Our survey results indicate that 63% of participants were willing to coexist with community cats and 61% agreed or strongly agreed with the use of TNR and its variations. Some countries such as France, Spain, Austria, Portugal, and Italy approved the TNR method for controlling community cat populations in national laws [12]. Although the TNR method is not explicitly referenced in any legislation in the United Kingdom or the United States, TNR programs are implemented by animal welfare organizations. The demonstrated benefits of TNR include reductions in nuisance complaints and cat populations in focal areas [33,41,45,46].

Nevertheless, the efficacy of TNR in achieving its population control objectives is contingent upon intensive and continuous TNR [47,48]. Boone et al. [49] used a stochastic simulation model to assess the impact of different management methods on community cat mortality over ten years. The researchers examined seven management scenarios, including (1) taking no action, (2) low-intensity removal, (3) high-intensity removal, (4) low-intensity episodic culling, (5) high-intensity episodic culling, (6) low-intensity TNR, and (7) high-intensity TNR. They further defined the outcome variable “preventable deaths” as the number of kitten deaths and lethal removal of adults because these numbers have the potential to be reduced by specific management actions. The simulation results indicated that the lowest cumulative number of preventable deaths over ten years for an initial population of 50 cats was observed in the high-intensity TNR scenario. In all management scenarios that Boone et al. tested, including removal and culling, the model predicted a reduction in the number of preventable deaths compared to a no-action scenario.

In addition to high-intensity and continuous TNR, friendly/socialized community cats may also be adopted [47,50]. For example, a two-year TNR program in a region of historically high cat impoundments in a Florida community, coupled with adopting socialized cats and nuisance resolution counseling for residents, has effectively reduced shelter cat intake [51].

In China, attention to community cats has arisen in community governance from the practical challenge of controlling their population to avoid residents’ complaints about noise and hygiene nuisances. The TNR method has garnered considerable support on social media in China [52]. Yet, solid empirical research demonstrating its effectiveness in reducing the population and increasing the welfare of community cats in urban China is still lacking. In 2007, researchers conducted a review of the feasibility of implementing TNR programs in Beijing [53]. They concluded that implementing TNR programs, in contrast to sending community cats to shelters, could be cost-effective, control community cat reproduction, be more humane than euthanasia in the shelters, and help strengthen disease prevention. The authors proposed that Beijing was well suited to TNR implementation and recommended government intervention in TNR programs. In 2019 and 2020, researchers at the Shanghai Animal Epidemic Prevention and Control Center reviewed the use of TNR in urban communities [54,55] and conducted a pilot study to evaluate the implementation of TNR in an urban community in Shanghai [56]. They found that implementing TNR over four months had significantly mitigated the surge in community cat populations and reduced noise disturbances in the community, and the environmental hygiene of the community had also notably improved [56]. However, they pointed out that capturing all community cats posed considerable challenges. To address these challenges, they recommended increasing financial support and public awareness, enhancing volunteer training, and strengthening research and technical support for TNR programs.

According to Article 30 of the revised Animal Epidemic Prevention Law of the People’s Republic of China (2021) (http://www.npc.gov.cn/zgrdw/englishnpc/Law/2009-02/20/content_1471591.htm, accessed on 4 August 2024), sub-district offices and township-level people’s governments are responsible for organizing and coordinating with residents’ committees and villagers’ committees to manage and handle stray dogs and cats within their jurisdictions to prevent the spread of diseases. Beyond this law, China does not have additional legal provisions explicitly addressing the welfare and protection of community cats and companion animals. Based on this law, Shanghai City has implemented TNR and adoption programs in communities supported by governmental agencies to regulate the population of community cats. Some first- and second-tier Chinese cities (e.g., Beijing and Hangzhou) have also implemented TNR combined with adoption programs within communities and city-wide adoption projects. However, these programs do not involve the participation and support of multiple government agencies, unlike the TNR programs in Shanghai. News reports indicate that the Center for Disease Control and Prevention [57], the Office of Spiritual Civilization Construction Committee [57], local sub-district offices and agricultural committees [58], and residents’ committees [37] have provided guidance or/and participated in the implementation of TNR programs in communities in Shanghai. For example, the Minhang District Government of Shanghai, through its “Today Minhang” app, offers ten free basic neutering/spaying packages each month for community cat rescuers/caregivers to apply for, including procedures such as health examinations, deworming, and vaccinations [59]. In the nearly two months since its implementation, the “Minhang District Stray Cat Neutering/Spaying Plan” has cumulatively served nearly 2300 community cats [60]. Residents in a community in Shanghai expressed high satisfaction with TNR programs, which significantly reduced noise complaints caused by community cats and effectively raised awareness of responsible pet ownership (e.g., preventing the abandonment of pets) [61].

Consequently, when coupled with adopting friendly/socialized cats, continuous TNR programs represent a viable method for controlling community cat populations in Chinese urban communities. Although both require high treatment rates to be implemented to reduce the population of community cats, TNR requires lower treatment effort than euthanasia [48]. In the absence of legislation to protect the welfare of unowned cats as well as companion animals, lethal methods such as trap-and-kill, culling, or euthanasia may be misused or abused by individuals or organizations under the guise of “animal welfare” or “animal management”. Our survey revealed that a substantial 71% of urban residents in China opposed the trap-and-kill method of managing community cats. This opposition aligns with the “no-kill movement” in the United States. It reflects two ethical theories: zoocentric ethics, which recognizes the intrinsic values of non-human animals, and virtue ethics, which validates compassionate considerations in decision making regarding community cat management [62]. TNR combined with adoption is more likely to garner broad public support and be effectively implemented to control the population of community cats in China.

In addition to evaluating the feasibility and effectiveness of TNR and its variations as well as lethal and non-lethal methods for controlling the population of community cats, it is crucial to adopt a multifaceted management approach. Integrated plans that focus on reducing uncontrolled breeding and preventing abandonment in the pet industry and within the owned cat population are also essential complements of any management strategies targeting community cats.

### 4.2. Educational Approaches

Regarding the attitudes towards managing community cats, we observed significant differences in participants’ gender and socio-economic status (SES, e.g., education and income levels). Male participants were more inclined to support trap-and-kill and taking no action and oppose TNR and centralized management methods than female participants. These results align with previous findings that female students and community cats interacted with each other more positively than male students at universities, and the density of male versus female students affected the dispersal, survival, and reproduction of community cats [5]. The gender differences in attitudes towards community cat management may be attributed to females’ greater empathy with animals and humans than males [63]. Furthermore, our findings indicate that residents with lower levels of education were more inclined to support trap-and-kill and taking no action than those with a university or college level of education. The relatively low-income participants were more inclined to support trap-and-kill and less inclined to support the TNR method, and they held more negative attitudes toward community cats than the relatively high-income participants. Our findings are consistent with those of previous studies in underscoring the importance of engaging with residents with low SES or from socio-economically deprived areas to address the overpopulation of community cats and improve their welfare [4,6,64]. Thus, in Chinese urban areas, the residents’ gender and SES influence their attitudes towards managing community cats, which may lead to disparities in the welfare of community cats and human–cat interactions.

The above findings provide valuable insights into tailored strategies for conducting educational campaigns effectively. The findings regarding gender and SES differences suggest that males, the relatively less educated, or/and the relatively less affluent may be the targets of educational campaigns to inform them about more ethical and effective methods of managing community cats, such as TNR combined with adoption in urban areas. As our findings also indicate that residents in fourth-tier cities are more likely to perceive the community cats as an unsolved issue and less inclined to support the use of trap-and-kill than their counterparts in first-tier or second-tier cities, educational campaigns in fourth-tier cities should focus on providing hands-on learning experiences about TNR techniques and fostering a culture of responsible pet ownership. In contrast, educational campaigns in first-tier and second-tier cities should focus on providing knowledge about alternative, non-lethal, and non-cruelty methods to manage community cats. Furthermore, providing accessible resources such as brochures, online guides, and videos on TNR procedures and responsible feeding practices can empower residents with the knowledge and tools necessary to participate actively in cat management efforts.

As humans create the problems of community cats, educational effort is a central component of any solution. Education, TNR, and adoption were determining factors in decreasing community cat populations over time [65]. Comprehensive education campaigns are essential to inform residents about responsible pet ownership (e.g., preventing abandonment and improving the welfare of pets), the intrinsic value of animals, the regulation of domestic cats’ reproduction (e.g., the importance of neutering/spaying), and the broader ecological implications of unmanaged cat populations. Those who hold neutral or undecided attitudes towards community cats and management methods can be empowered to shift their opinions and knowledge through outreach and education, potentially leading to an increased awareness of and commitment to the welfare of community cats.

### 4.3. Attitudes, Practices, and Scientific Findings

Public attitudes, perceptions, and management practices about community cats do not necessarily align with scientific findings, especially when these findings are inconsistent or contradictory. For example, in the current study, we examined public attitudes and perceptions regarding the role of community cats in rodent management. Scientific findings on this topic are mixed. A study conducted in Madrid, Spain, found that water sources, cat feeding stations, and green zones are significantly related to rat proliferation, indicating a relationship between these environmental features and the presence of nearby rats [66]. However, studies reporting the numbers of birds and rodents killed by cats fail to demonstrate how populations of these animals would change in the absence of cats or how rat densities could increase without feline presence [20]. Additionally, rats and other rodents can have more detrimental effects on biodiversity loss than cats [67,68], and the use of rodenticides can severely harm biodiversity [69,70]. Instead, attitudes and perceptions are socially constructed and shaped by multiple factors [71], including the socio-demographic variables we examined in this study.

Similarly, regarding the influences of TNR on the welfare and health conditions of community cats, previous scientific findings are mixed [51,72,73,74,75]. These inconsistencies may be due to variations in the implementation of TNR programs across different countries and regions. Factors such as whether the TNR program is intensive and continuous or a one-time event, whether it includes vaccination and adoption, whether it involves responsible feeding and regular medical checks for the neutered/spayed cats, and whether the program is implemented in an open, non-residential, tourist area or a residential, confined area can all contribute to differing outcomes. Additionally, previous studies rarely considered the welfare and health conditions of community cats before the implementation of a TNR program. Poor initial welfare and health conditions may result in no improvement or even deterioration in health after neutering/spaying. Therefore, we call for more research to consider the various variables in TNR programs and discuss the boundary conditions for the findings. Rather than drawing general conclusions about whether the TNR is effective or ethical, future research should aim to identify the specific conditions under which TNR programs are most beneficial for both the welfare of cats and residents.

Moreover, education plays a crucial role in bridging the gap between scientific understanding of the methods to manage community cats (e.g., TNR) and their practices in urban areas. A disconnection between scientific knowledge and the public’s understanding can impede the formulation and implementation of effective community cat management legislation. By integrating scientific knowledge with community-based education initiatives, we can create a more informed and compassionate society that supports the welfare of cats and residents.

### 4.4. Limitations and Future Directions

While this study provides valuable insights into public attitudes towards community cats and their management in urban China, three limitations should be acknowledged.

First, residents’ perspectives influence attitudes towards community cats and management strategies, which can vary significantly based on their affiliations with conservationist groups or animal welfare advocates. Those who advocate for the conservation of wildlife often highlight the impact of cat predation on native species, advocating for methods such as the total exclusion of domestic cats from natural environments, the containment of pet cats, the prohibition of outdoor feeding sites, or/and the cessation of TNR programs [22,76,77]. In contrast, animal welfare advocates, often including humane organizations, activists, and pet owners, tend to support management methods like TNR, particularly when they are coupled with adopting friendly/socialized cats [7,26,27,50]. Future research should investigate the influences of anthropogenic factors on attitudes towards cats and their management. For example, they may examine residents’ knowledge about cat welfare and predation behaviors, involvement in caretaking practices for community cats, and pre-existing interactions and attachments with pets.

Second, the study asked residents whether they considered community cats an unsolved issue without defining the meaning of “issue”. The “issue” in Chinese may be ambiguous. For example, does “issue” refer to the survival issue of community cats, such as the cats’ poor welfare and the need for improvement? Does it refer to the negative impact of community cats on the community environment, such as public health concerns and disputes among residents? Alternatively, does it refer to the lack of appropriate management methods for cats at a community level? The lack of a precise and clear definition of “issue” makes it difficult to explain why older adults, compared to younger adults, are more likely to view community cats as an issue while also more willing to coexist with them. It is also difficult to comprehend why residents of fourth-tier cities are more likely than second-tier cities to perceive community cats as an issue and less inclined to support trap-and-kill. Future research needs to explore the diverse interpretations of the “community cat issue” across various dimensions.

Finally, the current study employed a quantitative methodology, thereby precluding an investigation into the underlying reasons for the participants’ attitudes and their levels of understanding regarding cat management. Moreover, the subsequent options following the question about the willingness to coexist with community cats aggregate multiple elements into a single option. These elements include willingness to frequently see community cats and the hope that the cats are appropriately managed. The combination of multiple elements in one option may limit the ability to discern nuances in participants’ opinions about their coexistence with community cats. Therefore, qualitative research methods, such as interviews or case studies, may offer a more nuanced understanding of the factors influencing public attitudes towards community cats and their management. It is essential that management practices and regulations concerning community cats can be justified based on ethical and evidence-based perspectives, in addition to public attitudes.

## 5. Conclusions

This study sheds light on the complex landscape of public attitudes towards community cats and their management strategies in urban China, yielding two primary findings. First, a substantial proportion of residents were willing to coexist with community cats, voiced opposition to the trap-and-kill method, and expressed support for TNR and its variations. Considering the findings alongside existing TNR practices and legislation in China, we propose that TNR combined with adoption represents the most viable strategy for managing community cats in urban areas. However, measures aimed at curbing breeding and preventing the abandonment of companion animals are also indispensable. Second, socio-demographic factors such as age, gender, education, and income significantly influence attitudes towards different management approaches. Older residents, those with higher incomes, or residents in fourth-tier cities were more likely to endorse coexistence with community cats than younger or low-income residents or residents in second-tier cities. Higher socio-economic status (e.g., higher education or income levels) predicted more favorable attitudes towards community cats or stronger opposition to trap-and-kill or doing nothing as a management method. Males tended to support trap-and-kill and doing nothing while opposing centralized management and TNR methods, relative to females. These findings emphasize the importance of tailored educational campaigns to advocate for humane and effective management strategies, addressing both public concerns and the welfare of community cats. This study enriches the discourse surrounding urban community cat management, offering valuable insights for policy makers, advocates, and stakeholders dedicated to enhancing the welfare of both human and feline populations in urban China.

## Figures and Tables

**Table 1:** The frequencies, percentages, means, and standard deviations of the variables.

Variables	Response Categories	Frequency	Percentage (%)	*M*	*SD*
Willingness to Coexist with Community Cats (N = 5382)	Willing to coexist	3413	63	NA	
	Unwilling to coexist	774	14		
	I have not considered this issue.	1195	22		
Awareness of Community Cats as an Unsolved Issue (N = 4621)	Yes	2644	57	NA	
	No	1977	48		
Degree of Urgency of the Issue (N = 2644)	Not at all	112	4.2	3.38	1.03
	Slightly	281	11		
	Moderately	1206	46		
	Very	570	22		
	Extremely	475	18		
Management Method: Take No Action (N = 5382)	Strongly agree	429	8.0	3.38	1.15
	Agree	589	11		
	Neither agree nor disagree	1946	36		
	Disagree	1363	25		
	Strongly disagree	1055	20		
Management Method: Trap and Kill (N = 5382)	Support	272	5.1	NA	
	Oppose	3836	71		
	Neither support nor oppose	1274	24		
Management Method: Centralized Management (N = 5382)	Strongly agree	1517	28	2.24	1.05
	Agree	1780	33		
	Neither agree nor disagree	1533	29		
	Disagree	355	6.6		
	Strongly disagree	197	3.6		
Management Method: TNR and Its Variations (N = 5382)	Strongly agree	1507	28	2.25	1.06
	Agree	1795	33		
	Neither agree nor disagree	1509	28		
	Disagree	350	6.5		
	Strongly disagree	221	4.1		

Note: The *M*s and *SD*s in Table 1 were computed from the original scores of the 5-point Likert scale, with the responses “strongly agree” scoring 1 and the responses “strongly disagree” scoring 5. NA = not applicable.

**Table 2:** The frequencies (percentages), means, and standard deviations of items in the Attitudes towards Community Cats Scale (ACCS), N = 5382.

	Strongly Agree	Agree	Neither Agree nor Disagree	Disagree	Strongly Disagree	*M*	*SD*
It gives me pleasure to see or interact with healthy and lively community unowned cats outdoors.	1367	1451	1938	359	267	2.39	1.09
	(25%)	(27%)	(36%)	(6.7%)	(5.0%)		
Unowned cats add life and vitality to the community.	1151	1549	1887	502	293	2.49	1.09
	(21%)	(29%)	(35%)	(9.3%)	(5.4%)		
The existence of unowned cats in the community enriches the urban ecology.	1069	1431	1998	527	357	2.57	1.11
	(20%)	(27%)	(37%)	(9.8%)	(6.6%)		
Community unowned cats control rat populations and reduce the spread of disease.	1224	1778	1761	379	240	2.37	1.05
	(23%)	(33%)	(33%)	(7.0%)	(4.5%)		
Utilizing community unowned cats for ecological rodent suppression is better than dropping rodenticides.	1279	1686	1694	430	293	2.40	1.10
	(24%)	(31%)	(32%)	(8.0%)	(5.4%)		
Community unowned cats can protect old buildings and artifacts from rats.	1125	1590	1947	463	257	2.47	1.06
	(21%)	(30%)	(36%)	(8.6%)	(4.8%)		
Community unowned cats in heat can make noise and disrupt sleep.	803	1184	2323	725	347	2.75	1.07
	(15%)	(22%)	(43%)	(14%)	(6.5%)		
Community unowned cats can attack people.	275	518	2133	1494	962	3.44	1.05
	(5.1%)	(9.6%)	(40%)	(28%)	(18%)		
Community unowned cats can prey on birds.	290	674	2693	1120	605	3.20	0.98
	(5.4%)	(13%)	(50%)	(21%)	(11%)		
Community unowned cats are prone to harboring bacteria and parasites that affect human health.	601	1258	2205	773	545	2.89	1.10
	(11%)	(23%)	(41%)	(14%)	(10%)		

Note: The *M*s and *SD*s in Table 2 were computed from the original scores of the 5-point Likert scale, with the responses “strongly agree” scoring 1 and the responses “strongly disagree” scoring 5.

**Table 3:** Results of multilevel logistic regression models examining the effects of socio-demographic and community variables on residents’ willingness to coexist with community cats and awareness of community cats as an unsolved issue, with the answers “no” as references.

		Willingness to Coexist with Community Cats	Awareness of Community Cats as an Unsolved Issue
		Model 1, N = 4187	Model 2, N = 4621
		OR [95%CI]	OR [95%CI]
	Fixed effects		
	Intercept	0.02 ** [0.002, 0.25]	1.46 [0.15, 14.12]
Socio-demographic Variables			
Age	18–85	1.01 ** [1.00, 1.02]	1.02 *** [1.01, 1.02]
Gender	Female (ref.)		
	Male	1.19 † [1.00, 1.41]	0.89 [0.78, 1.01]
Education	College or university (ref.)		
	Elementary school or below	1.42 [0.95, 2.11]	0.75 † [0.56, 1.00]
	Junior high school	1.00 [0.76, 1.33]	0.97 [0.78, 1.20]
	High school	0.82 [0.66, 1.03]	1.01 [0.85, 1.20]
	Graduate or beyond	0.78 [0.55, 1.09]	0.85 [0.66, 1.09]
Employment	Full employment (ref.)		
	Part-time employment	1.05 [0.72, 1.52]	1.06 [0.79, 1.41]
	Household work	1.22 [0.87, 1.71]	0.94 [0.73, 1.22]
	Full-time student	0.96 [0.63, 1.47]	1.15 [0.88, 1.52]
	Unemployment	1.18 [0.73, 1.91]	0.88 [0.62, 1.24]
	Retirement	1.48 * [1.07, 2.04]	1.03 [0.80, 1.33]
Personal Income in the Last Year	<CNY 50,000 (ref.)		
	CNY 50,000–CNY 100,000	0.98 [0.80, 1.20]	0.90 [0.76, 1.05]
	CNY 100,000–CNY 200,000	1.43 * [1.06, 1.93]	0.85 [0.68, 1.05]
	>CNY 200,000	1.75 * [1.12, 2.74]	0.97 [0.72, 1.31]
Marital Status	Married (ref.)		
	Unmarried	1.89 *** [1.37, 2.61]	0.69 *** [0.56, 0.86]
	Divorced or widowed	0.72 [0.48, 1.07]	1.27 [0.92, 1.77]
Community Variables			
Type of City	Fourth-tier cities (ref.)		
	First-tier city	0.81 [0.53, 1.23]	0.59 * [0.39, 0.90]
	Second-tier cities	0.56 ** [0.37, 0.84]	1.22 [0.78, 1.91]
	Third-tier cities	0.75 [0.55, 1.03]	1.28 [0.92, 1.77]
Type of Community	Ordinary commercial housing community (ref.)		
	Old town community	1.29 [0.88, 1.88]	1.34 [0.91, 1.99]
	Indemnificatory housing community	2.93 * [1.05, 8.14]	0.55 [0.23, 1.37]
	Luxury housing community	0.96 [0.43, 2.12]	1.46 [0.60, 3.55]
	Urban village community	0.99 [0.69, 1.42]	1.05 [0.72, 1.53]
	Campus community	0.76 [0.29, 1.99]	0.61 [0.22, 1.64]
	Random effects		
	Intercept	0.10 * [0.05, 0.23]	0.16 *** [0.09, 0.28]

Note: OR, odds ratio; CI, confidence interval; † *p* = 0.048 or 0.047, * *p* < 0.05, ** *p* < 0.01, *** *p* < 0.001.

**Table 4:** Results of multilevel linear regression models examining the effects of socio-demographic and community variables on residents’ attitudes towards community cats and strategies for managing community cats, N = 5382.

		Attitudes towards Community Cats	Trap and Kill	Take No Action	Centralized Management	TNR and Its Variations
		Model 3	Model 4	Model 5	Model 6	Model 7
		*β* [95% CI]	*β* [95% CI]	*β* [95% CI]	*β* [95% CI]	*β* [95% CI]
	Fixed effects					
	Intercept	3.35 *** [3.23, 3.47]	1.29 *** [1.20, 1.39]	2.68 *** [2.48, 2.88]	3.68 *** [3.50, 3.86]	3.68 *** [3.50, 3.87]
Socio-demographic Variables						
Age	18–85	0.001 [−0.002, 0.003]	−0.002 [−0.003, <0.001]	<0.001 [−0.004, 0.004]	0.003 [<0.001, 0.01]	0.002 [−0.002, 0.005]
Gender	Female (ref.)					
	Male	−0.03 [−0.07, 0.001]	0.14 *** [0.11, 0.17]	0.09 ** [0.02, 0.15]	−0.11 *** [−0.17, −0.05]	−0.17 *** [−0.23, −0.11]
Education	College or university (ref.)					
	Elementary school or below	−0.01 [−0.09, 0.07]	0.13 *** [0.05, 0.20]	−0.002 [−0.15, 0.14]	0.14 * [0.01, 0.27]	0.08 [−0.06, 0.21]
	Junior high school	0.004 [−0.06, 0.06]	0.12 *** [0.06, 0.17]	0.14 * [0.04, 0.25]	0.05 [−0.05, 0.15]	−0.02 [−0.12, 0.08]
	High school	−0.01 [−0.06, 0.04]	0.08 *** [0.04, 0.13]	0.12 ** [0.03, 0.21]	0.01 [−0.07, 0.09]	−0.07 [−0.15, 0.01]
	Graduate or beyond	−0.03 [−0.10, 0.04]	0.06 [−0.003, 0.12]	−0.07 [−0.20, 0.06]	0.002 [−0.12, 0.12]	0.09 [−0.03, 0.21]
Employment	Full employment (ref.)					
	Part-time employment	−0.01 [−0.09, 0.07]	0.03 [−0.05, 0.10]	−0.02 [−0.17, 0.12]	−0.07 [−0.20, 0.06]	−0.03 [−0.17, 0.10]
	Household work	−0.01 [−0.08, 0.07]	−0.02 [−0.09, 0.04]	−0.15 * [−0.28, −0.01]	−0.10 [−0.22, 0.02]	−0.11 [−0.24, 0.01]
	Full-time students	0.02 [−0.05, 0.10]	−0.08 * [−0.15, −0.02]	−0.13 [−0.27, 0.004]	−0.09 [−0.21, 0.03]	−0.02 [−0.15, 0.11]
	Unemployment	−0.01 [−0.10, 0.09]	−0.01 [−0.10, 0.07]	0.06 [−0.12, 0.23]	0.01 [−0.15, 0.17]	−0.05 [−0.21, 0.11]
	Retirement	−0.01 [−0.08, 0.07]	0.02 [−0.04, 0.09]	−0.15 * [−0.28, −0.02]	0.03 [−0.08, 0.15]	0.07 [−0.05, 0.19]
Personal Income in the Last Year	<CNY 50,000 (ref.)					
	CNY 50,000–CNY 100,000	0.01 [−0.03, 0.06]	−0.03 [−0.07, 0.01]	−0.09 * [−0.17, −0.01]	0.01 [−0.06, 0.08]	0.05 [−0.02, 0.13]
	CNY 100,000–CNY 200,000	0.07 * [0.01, 0.13]	−0.10 *** [−0.15, −0.04]	−0.10 [−0.21, 0.01]	0.06 [−0.04, 0.16]	0.19 *** [0.09, 0.30]
	>CNY 200,000	0.13 ** [0.04, 0.21]	−0.11 ** [−0.19, −0.03]	0.02 [−0.13, 0.18]	−0.01 [−0.15, 0.13]	0.12 [−0.03, 0.26]
Marital Status	Married (ref.)					
	Unmarried	0.16 *** [0.10, 0.22]	−0.02 [−0.07, 0.03]	−0.13 * [−0.24, −0.03]	−0.05 [−0.15, 0. 05]	0.09 [−0.01, 0.19]
	Divorced or widowed	0.09 [−0.001, 0.18]	−0.06 [−0.14, 0.03]	−0.05 [−0.21, 0.12]	−0.02 [−0.17, 0.13]	−0.01 [−0.16, 0.15]
Community Variables						
Type of City	Fourth-tier cities (ref.)					
	First-tier city	−0.09 [−0.23, 0.04]	0.11 * [0.02, 0.21]	0.02 [−0.17, 0.20]	−0.31 *** [−0.48, −0.15]	−0.07 [−0.24, 0.10]
	Second-tier cities	−0.04 [−0.18, 0.11]	0.12 * [0.02, 0.22]	−0.002 [−0.20, 0.20]	−0.14 [−0.31, 0.04]	−0.07 [−0.24, 0.11]
	Third-tier cities	−0.08 [−0.18, 0.03]	0.04 [−0.03, 0.11]	−0.01 [−0.15, 0.14]	0.04 [−0.09, 0.16]	−0.03 [−0.16, 0.10]
Type of Community	Ordinary commercial housing community (ref.)					
	Old town community	0.02 [−0.11, 0.15]	−0.04 [−0.12, 0.05]	0.12 [−0.05, 0.29]	0.14 [−0.01, 0.29]	0.03 [−0.13, 0.18]
	Indemnificatory housing community	0.07 [−0.23, 0.37]	−0.05 [−0.25, 0.15]	−0.33 [−0.73, 0.06]	0.11 [−0.24, 0.46]	0.25 [−0.11, 0.61]
	Luxury housing community	−0.21 [−0.50, 0.09]	0.08 [−0.11, 0.27]	−0.05 [−0.43, 0.32]	−0.19 [−0.52, 0.14]	−0.20 [−0.54, 0.15]
	Urban village community	−0.04 [−0.17, 0.08]	−0.03 [−0.11, 0.06]	−0.06 [−0.22, 0.10]	0.12 [−0.02, 0.27]	0.10 [−0.05, 0.25]
	Campus community	−0.07 [−0.39, 0.26]	−0.02 [−0.24, 0.20]	−0.19 [−0.63, 0.24]	0.40 * [0.01, 0.78]	0.26 [−0.14, 0.65]
	Random effects					
	Intercept	0.02 *** [0.01, 0.03]	0.01 ** [0.004, 0.01]	0.03 ** [0.01, 0.05]	0.02 ** [0.01, 0.04]	0.02 ** [0.01, 0.04]

Note: *β* is the standardized regression coefficient; CI, confidence interval; * *p* < 0.05, ** *p* < 0.01, *** *p* < 0.001.

## Data Availability

The data presented in this study are available on reasonable request from the corresponding author.

## Appendix A. The Questionnaire Items in the Study

1. Which community do you currently live in? [Single-choice question]
  ○ The name of the community (this community)
  ○ Other community within the city where this community is located
  ○ Other community outside of the city where this community is located
2. What is your community type? [Single-choice question]
  ○ Old town community
  ○ Indemnificatory housing community (e.g., low-rent housing, public rental housing)
  ○ Ordinary commercial housing community
  ○ Luxury housing community (e.g., villa area, high-class residential area)
  ○ Urban villages community (e.g., urban community recently transformed from rural community, “villages in city”)
  ○ Other _________________
3. What is your gender? [Single-choice question]
  ○ Male
  ○ Female
  ○ Other
4. What is your age? (Please fill in a whole number, e.g., 26.) [Fill in the blank]
5. What is your current highest level of education (including those you are currently enrolled in)? [Single-choice question]
  ○ Below elementary school
  ○ Elementary school
  ○ Junior high school
  ○ High school
  ○ College or university
  ○ Graduate or beyond
6. What is your current employment? [Single-choice question]
  ○ Full employment
  ○ Part-time employment
  ○ Household work (refers to staying at home to take care of other family members, commonly known as “housewife” or “househusband”)
  ○ Full-time student
  ○ Unemployment
  ○ Retirement
7. Your personal gross income for 2020 is approximately? [Single-choice question]
  ○ Less than CNY 10,000
  ○ CNY 10,000–CNY 20,000
  ○ CNY 20,000–CNY 50,000
  ○ CNY 50,000–CNY 100,000
  ○ CNY 100,000–CNY 200,000
  ○ CNY 200,000 and above
8. What is your current marital status? [Single-choice question]
  ○ Unmarried
  ○ Married
  ○ Divorced
  ○ Widowed
9. Do you think unowned cats in your community have become an issue that needs to be solved? [Single-choice question]
  ○ Yes
  ○ No (Please skip the Question 10)
10. With 5 being extremely urgent and 1 being not urgent at all, how urgent do you think it would be to solve the issue of unowned cats in your community? [Single-choice question]
  ○ 1
  ○ 2
  ○ 3
  ○ 4
  ○ 5
11. What is your attitude towards the phenomenon of trapping and killing unowned cats in the community? [Single-choice question]
  ○ Support
  ○ Oppose
  ○ Neither support nor oppose
12. What is your overall attitude towards unowned cats in the community? [Single-choice question]
  ○ I am willing to see unowned cats regularly in the community, hoping that they will become part of community life, be treated well, and be managed appropriately to fulfill their positive roles.
  ○ I am relatively willing to see unowned cats in the community in the hope that they can be managed scientifically.
  ○ I have not considered this issue.
  ○ I am less willing to see unowned cats in the community, but disagree that the community uses extreme methods to manage unowned cats.
  ○ I am totally unwilling to see unowned cats in the community.
13. Your opinions on unowned cats in the community: [Matrix single-choice question]. Strongly agree, agree, neither agree nor disagree, disagree, strongly disagree
  13-1. It gives me pleasure to see or interact with healthy and lively community unowned cats outdoors.
  13-2. Unowned cats add life and vitality to the community.
  13-3. The existence of unowned cats in the community enriches the urban ecology.
  13-4. Community unowned cats control rat populations and reduce the spread of disease.
  13-5. Utilizing community unowned cats for ecological rodent suppression is better than dropping rodenticides.
  13-6. Community unowned cats can protect old buildings and artifacts from rats.
  13-7. Community unowned cats in heat can make noise and disrupt sleep.
  13-8. Community unowned cats can attack people.
  13-9. Community unowned cats can prey on birds.
  13-10. Community unowned cats are prone to harbor bacteria and parasites that affect human health.
14. Your attitudes towards the following methods of management of unowned cats in the community: [Matrix single-choice question]. Strongly agree, agree, neither agree nor disagree, disagree, strongly disagree
  14-1. Take no action.
  14-2. Neuter/spay and vaccinate the cats after capture; treat, foster, return to the original location, or otherwise rehome them, as appropriate.
  14-3. Centralized management: After capturing the cats, send them to shelters or stray animal bases.
